# Using a Trauma-Informed Care Approach to Understand Family Caregivers’ Experiences of Accessing Formal Supports in Dementia Care

**DOI:** 10.1177/10748407251314549

**Published:** 2025-02-06

**Authors:** Christine Meng, Safira Lachapelle, Adebusola Adekoya, Lucy Kervin, Kishore Seetharaman, Koushambhi Basu Khan, Jennifer Baumbusch

**Affiliations:** 1 The University of British Columbia, Vancouver, Canada; 2 University of Alberta, Edmonton, Canada; 3 University of Waterloo, Ontario, Canada; 4 Simon Fraser University, Vancouver, British Columbia, Canada

**Keywords:** trauma-informed care, dementia, family caregivers, qualitative, formal supports

## Abstract

Family caregivers provide essential care and support for individuals living with dementia, yet their contributions and needs are often unrecognized within formal health care systems. Over time, this marginalization can contribute to long-term trauma. Guided by a trauma-informed care (TIC) framework, we explored the experiences of 15 family caregivers in a longitudinal, qualitative study. Set in British Columbia, Canada, data were collected through semi-structured interviews and reflective diaries. Data were analyzed using inductive-deductive thematic analysis. Deductive analyses demonstrated that participants’ experiences aligned with existing TIC principles. Inductive analysis identified “Uncertainty” as a novel principle, reflecting the ongoing challenges caregivers face from diagnosis to the inadequacy of in-home supports. Our study highlights the importance of recognizing trauma induced by interactions with formal health care services and the value of using a TIC approach with family caregivers of people living with dementia.

## Introduction and Background

Family and friend caregivers provide significant support for individuals living with dementia. Caregiving is defined as “the provision by a family care provider of appropriate personal and healthcare for a family member or significant other” (Swanson et al., 1997, p. 68). Family caregivers—including spouses or partners, children and children-in-law, grandchildren, and chosen, or non-biological, family—often co-reside with individuals living with dementia and assume physical, emotional, social, and financial responsibilities (Bressan et al., 2020). Care trajectories include both episodic and enduring patterns of care over the life course, leading to dynamic and variable caregiving experiences across families (Magnaye et al., 2020).

Substantial evidence indicates that providing care for people living with dementia has negative impacts for the physical, mental, social, and emotional well-being of caregivers, and these impacts may intensify over time with progression of the disease and subsequent increases in caregiving demands (Bouldin & Andresen, 2010). Due to gaps in care arising through a lack of adequate systemic formal supports, caregiving frequently requires families to commit significant amounts of time and energy to caregiving tasks. Caregivers may dedicate upward of 40 hr per week to care tasks (MacLeod et al., 2017) and support their family members for an average of more than 5 years, and in some cases, 10 or more years (National Alliance for Caregiving & American Association of Retired Persons, 2015). This intense commitment is linked to elevated stress levels and compromised health, which can lead to anxiety, fatigue, depression, and isolation among caregivers (Sinha, 2013; Statistics Canada, 2018; Van Wijngaarden et al., 2018). Caregivers also experience socioemotional repercussions and a sense of loss due to role and relationship changes arising through caregiving demands (Chiu et al., 2016).

The impacts of caregiving often extend beyond physical health. Burnout among family caregivers—who are most commonly women—often leads to their exiting the workforce prematurely, reduced work hours, and lower wages compared with non-caregivers (Keating et al., 2021). Longitudinal research from Canada shows that long-term, high-intensity caregivers are significantly more likely to be fully or partially retired and not participating in the labor force compared with non-caregivers (Zhang et al., 2022). Prematurely exiting the workforce involuntarily creates financial challenges for these caregivers and further exacerbates their mental health stress and isolation (Peacock et al., 2020; Zhang et al., 2022). Poor mental health outcomes resulting from caregiving experiences and processes are a significant concern for caregivers. Systematic reviews indicate that caregiving for individuals with dementia poses serious risks to the caregiver’s physical and mental health, as well as their overall well-being (Brini et al., 2022; Stall et al., 2019). The distress experienced by caregivers has been associated with declines in cognitive function, mood, quality of life, and functional abilities (Stall et al., 2019).

Although the issues faced by caregivers of people living with dementia in relation to inadequate formal supports, caregiving demands, and subsequent impacts on their well-being are well researched and understood, less research has specifically focused on how these factors contribute to this group’s experience of trauma, and the practices that may be leveraged to mitigate trauma-related repercussions among families. Studies on related topics in the dementia space have highlighted the potentially traumatic impact of caregivers’ experiences with people living with dementia, including relational loss (Asher & Starr, 2021; Bergman et al., 2016; Nathanson & Rogers, 2020). Previous research on the viewpoints of family caregivers suggests they experience vicarious distress and, in some cases, primary trauma as a result of caregiving itself (e.g., confronting the behaviors, thoughts, and emotions of an individual living with dementia) as well as interactions with the formal health care system (e.g., transitioning their family member to a care home) (McCormack et al., 2017; Robertson et al., 2024; Wharton & Ford, 2014). The Substance Abuse and Mental Health Services Administration (SAMHSA, 2014) defines trauma as

a single event, multiple events, or a set of circumstances that is experienced by an individual as physically and emotionally harmful or threatening and that has lasting adverse effects on the individual’s physical, social, and emotionally harmful and that has lasting adverse effects on the individual’s physical, social, emotional, or spiritual well-being. (p. 7)

Trauma can result from any experience perceived as a threat to one’s life or physical integrity, or the life or integrity of a close relative (Varghese & Emerson, 2022).

Health care professionals are tasked with responding to trauma-related needs (Reeves, 2015). However, caregivers seeking formal supports for those living with dementia often encounter a health care system that is ill-equipped to address their complex emotional and physical needs. Family caregivers frequently report feeling coerced into compliance with health care staff, with formal resources typically allocated only after persistent issues arise (Lilly et al., 2012; McCormack et al., 2017). This inadequacy is exacerbated by the challenges of navigating support services and the health care system’s failure to prevent caregiver burnout and decline. Due to gaps in services and supports, caregivers commonly assume additional caregiving responsibilities not covered within the formal system (Hazzan et al., 2022). Their practical, psychological, and emotional needs arising through these roles—such as feelings of loss and overload within the formal dementia care system—often go unaddressed in formal care settings (Boss, 2010; Vipperman et al., 2023). This has consequences for the well-being of caregivers and their ability to continue providing care.

## Theoretical Perspective

Trauma-informed care (TIC) is grounded in Trauma Theory (Kusmaul & Anderson, 2018; Reeves, 2015). According to this theory, “if traumatic memories cannot be verbally or symbolically processed, they are stored as physiological reactions to stimuli, situations, or states of arousal that recall the traumatic experience” (Reeves, 2015, p. 698). Unprocessed traumatic events can lead to unpredictable trauma symptoms and responses to stimuli unrelated to the original traumatic event (Reeves, 2015). Over the past 30 years, a range of innovative ideas have shaped the development of TIC concepts, which are now applied in various contexts including child welfare systems, schools, criminal justice facilities, and mental health and substance abuse treatment providers (Wilson et al., 2013).

Trauma-informed care is defined in this article as a strength-based framework that encompasses six principles outlined by SAMHSA (2014): safety; trustworthiness and transparency; peer support; collaboration and mutuality; empowerment voice and choice; and cultural, historical, and gender considerations. TIC has gained increasing attention across diverse care settings. Robertson et al. (2024) propose a framework for trauma-informed home visits, emphasizing the need for universal safeguards to prevent re-traumatization in older individuals. For example, being in what is normally a safe space for an older adult who has experienced care-related trauma may increase their vulnerability during clinician home visits. Implementing these safeguards, regardless of the setting, can enhance comprehensive care for older people (Robertson et al., 2024).

Despite the growing body of literature on TIC, limited studies have adopted a TIC lens to understand family caregivers’ experiences in accessing formal supports for dementia care within a Canadian community context (Isobel et al., 2021; Palfrey et al., 2019; Wilson et al., 2017). Unrecognized and unsupported needs of family caregivers within formal care systems carry significant consequences for individual well-being and, subsequently, demands for supportive services. It is important to consider the role of trauma in the caregiving experience to inform care practice and delivery for this population. Guided by a TIC framework, the purpose of this study was to explore the experiences of family caregivers of people living with dementia as they access formal support services. We aimed to address the following questions: (a) How is TIC evidenced in care interactions as family caregivers engage with formal support systems? and (b) What recommendations can be made to customize and adapt TIC practices for dementia care?

## Method

This study applied a longitudinal, qualitative approach involving repeated data collection with individuals over time to capture the temporality of phenomena (Derrington, 2019). This approach is appropriate for investigating the experiences of family caregivers of persons living with dementia as it may effectively capture complex health trajectories and care transitions, particularly for progressive conditions (Calman et al., 2013; Tuthill et al., 2020). Research ethics approval was obtained from the University of British Columbia’s Behavioural Research Ethics Board (REB #H20-01093).

### Setting

This study was conducted in British Columbia, the westernmost province of Canada. In British Columbia, home and community care services are governed, planned, and delivered by five regional health authorities overseen by provincial administration and legislation. This research was conducted against the backdrop of the global coronavirus (COVID-19) pandemic, which has been evidenced as having significant negative impacts on the well-being of persons providing and receiving familial care (Bergmann & Wagner, 2021; Budnick et al., 2021; Wister et al., 2022). Focusing on the experiences of caregivers of community-dwelling persons with dementia allowed us to home in on challenges unique to the navigation of and access to community-based care and supports.

### Recruitment and Participants

A purposeful criterion-based sampling strategy was used (Patton, 2014). To be eligible for participation, individuals were required to meet the following criteria: (a) primary caregiver for a community-dwelling person living with dementia, (b) residing in British Columbia, Canada, and (c) fluent in English. A range of recruitment strategies were applied and included advertisement on social media platforms, in the newsletters of community organizations and with the Alzheimer’s Society of British Columbia, along with word of mouth (e.g., referral by other participating caregivers or families). Following initial contact, participants were screened for eligibility by a research team member. The caregiver sample was comprised of three males and 12 females aged 36 to 83. They were responsible for caring for a person with dementia between 30% and 100% of the time, with a median responsibility of 95%. Relationships between participating caregivers and recipients included seven daughters, six spouses, one sibling, and one daughter-in-law. Employment statuses among the caregivers were as follows: 11 retired, one on leave, two full-time, and one part-time. Annual household incomes ranged from less than $20,000 to $200,000.

### Data Collection

Data for this study were collected as of a longitudinal qualitative study investigating the experiences of caregivers of persons with dementia as they provide care and navigate care systems over time. Over a 2-year period, caregivers participated in semi-structured interviews every 4 months and completed regular narrative diary entries (due to staggered recruitment, participants entered and exited the study at different points in time; in some cases, participation ended before the 2-year period due to the death of the person living with dementia or their transition into long-term care). Interviews were conducted between August 2020 and October 2023 by two research team members with expertise in dementia care and qualitative methods via the virtual platform Zoom or by telephone. Diaries were generally completed monthly but timing accommodated participant preferences and availability. Data collection continued until 2 years of collection were complete or until the death or transition of the person living with dementia to a long-term care home. Interview questions were both closed and open-ended and covered topics related to caregivers’ daily lives, interactions with the formal care system, and reflections on their caregiving role. Diaries were written in response to an open-ended question prompting description of participants’ general experiences and high and low points related to their caregiving role. Caregivers completed on average five interviews—ranging from 33 to 155 min in duration—and 11 diaries. Informed consent was attained from all participants prior to beginning of each data collection point.

### Data Analysis

A combined deductive-inductive qualitative analysis approach was applied to interpret caregivers’ experiences in accessing formal supports with individuals living with dementia. Data familiarization was performed by thoroughly reading interview transcripts and diary entries, gaining an understanding of each family’s journey. The six principles from the TIC framework were then applied to index passages deductively, adhering to a modified framework method (Ritchie & Spencer, 1994). Subsequently, indexed passages were organized, plotted for analysis, and reviewed with co-authors during the charting stage (Ritchie & Spencer, 1994). Through inductive analysis, the authors extracted sections from the data within each principle and identified recurrent patterns. Passages that did not align with the identified themes were independently reviewed by the authors to assess if they represented variations or additional themes. Collectively, the authors determined the final list of themes, selected exemplars, and constructed interpretations of caregivers’ experiences.

### Rigor

To address rigor, an audit trail of research team minutes and process memos was created (Onwuegbuzie & Leech, 2007). Furthermore, collaboration and reflective conversations within the research team during coding, along with the iterative building, review, and revision of themes, enhanced the team’s credibility (Onwuegbuzie & Leech, 2007). Triangulation of data from multiple sources (i.e., interviews and personal diary entries) was used to help validate the findings of the study.

## Findings

The findings are presented in alignment with the principles of TIC. Table 1 presents the original principles of the TIC framework (SAMHSA, 2014), their definitions, and how they may be interpreted in the context of community-based family caregiving for an individual living with dementia. Each principle serves as a lens through which we examine the caregivers’ experiences with accessing formal support for individuals with dementia. We use quotes to illustrate how each principle is reflected in their experiences, blending theoretical context with examples. We have also introduced an additional principle, “uncertainty,” to address the unique challenges associated with the unpredictable progression of dementia.

**Table 1.** TIC Principles in Dementia Caregiving.

Label	Original description	Adapted description	Clinical implications
Safety	Ensuring that patient and staff member safety is a top priority, both physically and psychologically	Creating an environment where caregivers feel physically and psychologically safe, while minimizing the risks of re-traumatization or harm through interpersonal interactions	Providing a space that is physically and psychosocially safe when family caregivers access support, including frequent check-ins through services, to assess the needs of both the caregiver and the individual living with dementia. Being able to acknowledge and actively address the family caregiver’s past experiences with interacting with the health care system could have a lasting impact on their overall well-being
Trust worthiness and transparency	The system operates in a transparent manner with the intention of enhancing and sustaining trust among and between all levels of the system (i.e., administrators, staff, and patients)	Establishing reliability, dependability, and transparency when caregivers access care entails practicing openness and clear communication. This encompasses ensuring that information, actions, decisions, and processes are easily accessible and understandable to all involved parties	Health care professionals should take the lead in finding alternative options when designated formal supports are missing. This includes providing updates in a timely manner and actively seeking input from family caregivers and individuals living with dementia, whether the needed services become available or unavailable
Peer support	Peer support and mutual self-help are key vehicles for establishing safety and hope, building trust, enhancing collaboration, and utilizing their stories and lived experience to promote recovery and healing	Connecting caregivers with individuals who have similar lived experiences provides access to emotional, informational, and practical assistance, fostering mutual self-help among those who share similar experiences	Investments in training health care professionals and trained facilitators in a peer support group, to possess basic knowledge of mental health first aid are crucial. Additionally, organizations should provide opportunities for caregivers to establish self-help groups within families that share similar experiences
Collaboration and mutuality	Recognition that all individuals in an organization have a role to play in creating and maintaining a trauma-informed system. Partnerships are emphasized in an attempt to level power differentials	Practicing power-sharing and collaboration involves a cooperative effort where caregivers, groups, or organizations work together to achieve a common goal. This fosters a sense of balance, reciprocity, and shared understanding in relationships or interactions, highlighting the importance of everyone’s role in the process	Adopting a relationship-based, trauma-informed approach plays a crucial role in the interactions between health care professionals, individuals, and their family caregivers. This approach also extends to the relationships between health care professionals, the broader community, and fellow health care practitioners. By fostering collaboration and mutual respect, this approach values the knowledge of various “care experts,” enabling a broader range of treatment options and plans
Empowerment voice, and choices	Strengths and experience are emphasized in an attempt to build resilience and promote healing. Shared decision-making is emphasized in the recovery process	Recognizing that each caregiver’s experience is unique necessitates an individualized approach. It also entails offering opportunities and resources for the target population to actively shape policy and address community-level concerns, while acknowledging their strengths and contributions	It is important to understand that each family’s journey through accessing formal support is unique, with its own strengths and limitations. Rather than applying a one-size-fits-all approach to all families seeking formal support, health care professionals should actively involve individuals and their caregivers in the decision-making process, including at different levels of policy-change meetings
Cultural historical and gender issues	Identity is recognized as a critical component of healing; leverages the healing value of traditional cultural connections; incorporates policies, protocols, and processes that are responsive to the racial, ethnic, and cultural needs of individuals served; and recognizes and addresses historical trauma	Actively moving beyond cultural, historical, and gender stereotypes and biases involves providing culturally responsive services for caregivers, harnessing the healing value of connections, and acknowledging as well as addressing trauma experienced by caregivers	Recognizing the emotional journey that individuals and their caregivers undergo is crucial. This journey may intersect with factors such as sex, gender, cultural background, and socioeconomic status. Health care professionals should actively address trauma experiences with integrated resources and continually practice self-reflection to examine their practices with individuals and family caregivers
Navigating uncertainty	N/A	Recognizing caregivers’ need to address uncertainty throughout their journey is crucial. Acknowledging their feelings and fostering skills to cope with ambiguity and adapt to changes, relying on the best available information, are essential when navigating uncertainty	Recognizing that there is no “ending” to a caregiver journey, and the family’s needs change along the journey, accompanies many feelings of uncertainty. Different organizations should partner to support the caregiving journey with family caregivers and individuals living with dementia, ensuring a smooth and multidirectional path for them to access support. Also, by acknowledging the temporality, organizations can offer families more opportunities to try out different services to address uncertainties in the journey

### Safety

Safety encompasses both physical and mental aspects of the various interactions that participants had within formal support systems. The absence of adequate services often creates situations that compromise participants’ physical and emotional safety. P10 (62-year-old daughter) expressed frustration about the chronic shortage of home care support. She highlighted staffing inadequacies, stating: “It’s always a critical shortage, nothing critical about it; this is a normal shortage! Hire people! Bring in outside help! Now I’m just ranting!” The persistent scarcity of essential resources, such as home care, was reported by many participants as leaving them with insufficient support and ultimately leading to detrimental physical and psychological outcomes.

The adequacy of home care, both in terms of availability and the training or willingness of health care workers to work with individuals living with dementia, was described a significant concern. As a result, participants often find themselves compensating for these deficiencies, frequently at the expense of their own physical and mental health. Many participants had pre-existing health conditions that required medical attention. The added responsibilities exacerbated these conditions, as P07 (72-year-old wife) noted in a diary entry: “I am at a point of despair. I was forgetting to take my pills . . . I have high blood pressure and Addison’s disease; stress worsens both conditions.” Similarly, P06 (43-year-old daughter) shared that stress from home care support scheduling impacted her sleep:

They could just swap hours between the regular and the replacement. It would be enough. Anyway, this stresses me out in a major way. However, after three nights without sleep over this, I finally convinced the scheduler to adjust, and she is giving me three days this week of a schedule that works for me.

Participants emphasized deteriorating mental health as a result of their interactions with the health care system. In particular, participants described how their mental health safety was compromised when their challenges went unrecognized by care providers during medical consultations. P12 (60-year-old daughter) explained in an interview the need for health care providers to acknowledge their mental well-being:

I often find that the medical community tends to be focused a lot on, understandably and that it’s important, the person being cared for. But the person providing the care, when you talk about being stressed and you talk about you know, sometimes feeling a bit overwhelmed. I remember I used to go to a drop-in clinic where I’d get my allergy shots and there was one very sympathetic doctor who really cared or would ask questions about me when I’d drop in for my shots once he knew I was a caregiver. And that’s so rare. Like even my family doctor, it’s not like, “Well how’s it going?” And I think it’s important that they ask that sometimes because sometimes it does get very difficult, especially when I was working and trying to cope with everything that was going on. (P12)

Similar experiences were shared by P07, who elaborated on a situation where only two questions were permitted during a doctor’s visit: “Okay, then, you go to the doctor, and you’re only allowed two questions. So, I have to phone back.” Due to time constraints and the limited number of questions allowed during medical encounters, participants are often placed in situations where they must prioritize their concerns and needs. As a result, questions and concerns related to their mental health and safety are deferred to address more immediate and time-sensitive care concerns.

### Trustworthiness and Transparency

Trustworthiness and transparency, or a lack of these concepts, were evident in participants’ interactions at organizational and interpersonal levels when they accessed formal support. At an organizational level, participants reported constant letdowns by the system in care planning. For instance, when interviewed about home care services not being on time, P10 stated, “This is not unusual. Oftentimes they [home care services] just don’t show up or they’ll call me ten minutes before to cancel.” Participants found themselves in a dilemma when planning due to the unpredictability of whether home care services would show up or not, ultimately leading to sacrifices in their personal space and time. P10 elaborated, “Here we go again anxiety about whether or not home support will show. I have no plans to meet anyone for the respite time because I hate to disappoint people.” P10 further explained this frustration: “What’s the point? . . . like I’m going to make plans and I’m going to break the plans. I’m getting a bad reputation with friends. ‘Oh, you know, she won’t show up, so don’t worry about it.’”

Participants also reported experiencing a lack of transparency within the health care system related to care coordination, often finding themselves taking the lead in seeking information and resources. This process was time-consuming and often accompanied by confusion and frustration. For example, P04 (82-year-old wife) described a situation in which she made several attempts to obtain a “piece of paper” from the adult day program coordinator to enroll her husband in the program. “I talked to [adult day program coordinator] again, that was about almost six weeks later and said, ‘Did you get the request for [adult day program enrolment]?’ She said, ‘No, I have nothing.’ So, I had to go through it again.” Participants took action to ensure care coordination for the needed support and resources; however, P04 expressed, “all those things should be made easier . . . not that you always have to phone and phone and phone and you don’t get any answers.” On the other hand, P09 (36-year-old daughter) praised a nurse’s action when the nurse actively acknowledged this participant’s feelings upon discovering that the referral for her family member had been lost in the system. P09 explained in her diary entry:

I decided to wait a day to decide what to do and then I got a call from—[who is a nurse who covers for the nurse assigned to my dad when she is off]—she had been by to see my dad and said he seemed confused . . . I explained the whole case manager thing to [nurse] who acknowledged how frustrated I was and said she would put in the referral right away—phew—finally someone who can actually help me! (P09)

This acknowledgment of frustration and unmet needs on an interpersonal level helped to build a trusting relationship between the participant and the health care team. In contrast, issues with trust and transparency traumatized participants when they witnessed changes in family members living with dementia while struggling to receive support. For instance, P07 recalled an appointment she thought was an assessment for a day program but was not. She said,

so much to my disappointment we have to keep fighting with [health authority] and hope for the best . . . [my husband] is losing his speech and his desire to be around people. It is very frustrating to watch this happen.

Trust and transparency in relationships between participants and health care providers were also compromised when health care providers were too disease-oriented, rather than person-and-family-oriented. P06 provided an example of her experience when she received the diagnosis for her mother:

I didn’t really . . . believe the doctor at first because it was such a horrible experience. I couldn’t trust her at all and I still don’t trust her . . . she did not have a full picture of what my mom was like, who my mom was, what our life was like, what her life was like. It was simply like, you sit down, you do this test in ten minutes and if you fail, you have dementia . . . there was no reason for me to trust this woman. (P06)

The health care provider’s failure to view the person with dementia holistically in relation to both their disease and personhood, along with a lack of consideration for how the diagnosis impacted both the participant and the entire family, posing significant challenges to building safe and trusting care relationships.

### Peer Support and Self-Help

Participants expressed the need for a space to share experiences and acknowledge feelings, either through a professionally led peer support group or a self-help group facilitated by caregivers with similar experiences. P07 articulated, “I felt the need to be alone, yet I was feeling lonely . . . I find all the stories I have made up to keep things calm disturbing. I feel so sorry for him too, as I know he does not know what to do with himself.” Due to the unavailability of services such as adult day programs because of pandemic-driven closures, participants often stepped into these roles, without the resources and supports to adequately meet the person with dementia’s needs. P07 further explained,

I told [the doctor] how overwhelmed I was getting with caring for P07A. All the programs had been shut back down; I could not provide the stimulation he needs on a daily basis. He needs his days to feel purposeful and that he has accomplished something.

In addition, participants highlighted the important role of facilitators in professionally led peer support groups in addressing or exacerbating trauma. The experiences shared by participants can be traumatic and have lasting impacts. P06 shared an instance where a group facilitator abruptly redirected attention away from her when she was visibly distressed while sharing her experiences:

That was a major problem with the [non-profit organization] . . . I’d had the facilitator cut me off when I’m crying and be like, “Okay, we’re going to need to move on to someone else now.” I was like, “Well you let the other person talk for 45 minutes and it’s not my fault we only have ten minutes left.” (P06)

This participant felt dismissed and unsupported, which compounded her stress and isolation. This inadequate acknowledgment of the participant’s emotional distress, particularly in the context of vulnerable disclosure, highlights a gap in trauma-informed training or practice within this formal support group setting.

Participants reported a desire to connect and share experiences with other caregivers, potentially through establishing self-help networks with other families. P08 (66-year-old sibling) emphasized the significance of understanding the experiences of other families on this journey:

Is this normal for families? They’re going to have all these highs and lows for it all . . . That’s what I haven’t seen in [non-profit organization’s] support . . . I’m sure they say every family’s different but are there some common aspects?

While each family’s caregiving journey for individuals with dementia is unique, participants expressed the need to form connections with other caregivers navigating similar paths. They felt that such connections could provide a shared lived experience and practical knowledge component lacking in professional support groups.

### Collaboration and Mutuality

In the context of dementia care, collaboration occurs among participants, program coordinators, health care professionals, and home care workers, among others. However, participants described attempting to partner with health care professionals, only to encounter resistance to power-sharing. For instance, P09 explained how important food preparation was for her father’s care, and how difficult it was for her to access support:

The . . . issue of having the nurse . . . do more than simply watch him take a pill was to have them try to do more simple food prep for him which he also needs, but again, I ran into road blocks every which way . . . just a nightmare of a headache—plus the fact that the nurses where adamant that they could only do very LIGHT meal prep—which I tried hard to decipher, I asked if there were any easy recipes I could shop for groceries for that the nurses could make and received an email back explaining that they couldn’t be responsible for making “recipes.” (P09)

In an effort to engage with nursing staff in a power-sharing process around food-planning and preparation, the participant made an active effort to shop for, plan, and track meals for her father. However, despite these efforts, they encountered “roadblocks” at every turn and a collaborative solution was not reached.

P06 had a similar experience when she inquired whether a small amount of dressing supplies could be stored at their home for easier access and management of wound care. She found the home care nursing team was “passive-aggressive” and would not share “80 cents worth of supplies.” Instead, the home care nursing team referred P06 to a distant pharmacy to purchase them. To collaborate and reach a mutually effective solution, P06 offered two potential options to the home care nursing team: (a) “give me extra home support hours to enable me to make that trip” or (b) “come every day to do the wound care.” Both options were met with “negative responses.” No further negotiation occurred, indicating a lack of power-sharing between the home care nursing team and the participant. These experiences exemplify an absence of collaborative efforts from health care providers despite participants’ attempts to find mutually beneficial options to achieve their goals.

### Empowerment, Voice, and Choice

Empowerment within formal supports from TIC approach would include opportunities for participants to shape policy or address community-level concerns as well as individual needs. At a community or policy level, factors such as the participants’ schedule, work obligations, time, and service costs did not appear to be part of formal services decision-making. P06 elaborates,

The system really isn’t set up to meet BC’s mandate of Home First . . . It’s so ridiculously limited . . . I need an advocate. Why do they make receiving care so challenging? We need to be able to design it ourselves. The gov needs to give us the money to hire our own help. (P06)

P05 (63-year-old husband) described this in another way, “it’s like saying, ‘Tell the truck how he needs to drive.’ You know what I mean? No, I’ve been there, done that.” Many participants had ideas about how policy could be shaped to improve their experience. For example, specialization created a problem where home care case managers were unable to answer questions that relate to service costs, scheduling, or day programs. While case managers were meant to be the “point person” they, in fact, referred participants to numerous other departments and people, creating a tangled web of information that participants had to navigate on top of their day-to-day obligations.

At an individual level, many participants expressed preferences for the services they would like to receive; however, they encountered challenges in meeting those needs. Many were frustrated and stressed from having to constantly accommodate the system and its requirements. P10 explained, “You have to work your schedule into theirs even though they’re supposed to be working to help you.” Participants who attempted to proactively assert their voice and preferences in care coordination, such as scheduling with home care services, like P06, were shut down. “I was informed on Wed—only because I asked—that the regular worker would start her old shifts on Sunday. My days are jam packed with work and caregiving things and they have given me zero notice.” The concept that formal services exist to support families was challenged by these lived experiences, as participants’ preferences and needs were often not prioritized when they conflicted with the types of support the health care system offers. Participants were expected to modify their expectations to accommodate the health care system to receive, or partially receive, the support they requested.

Care-related preferences (meal planning, transportation, treatment planning, daily tasks, communication) were discounted at the individual level and created traumatic conditions for participants. P07 described feeling like she was “walking on eggshells,” bearing numerous responsibilities to compensate for the lack of formal support for the individual living with dementia. P07 expressed a need for human connection and was offered counseling; however, the sessions were only virtual, a communication method that did not work for her and was not her preference. Participants expressed their need for support and described the negative emotions they experienced when those needs were not met, leaving them feeling powerless and deprived of voice and choice.

### Cultural, Historical, and Gender Issues

Generationally, there were varying degrees of trust and acceptance of the health care system among participants. For example, some participants were more culturally rooted in a biomedical model of care that regarded health care professionals as experts who “know best.” As P09 explained in an interview while trying to negotiate home care supports for her father residing at home, “He won’t accept the help unless he’s told by some authority on high that it is needed, but this organization won’t give the help unless he acknowledges that he needs it, and I feel stuck in the middle.” The rigid process for accessing home supports does not clearly apply in this situation, placing this participant in a dilemma where the guidelines were not set for considering sociocultural norms and expectations influencing acceptance of care.

Although there was limited representation of male participants in this study, P09’s perspective of her father’s experience demonstrated how gender may play a role in how care is planned and delivered for people living with dementia: “ . . . they see all the presence of [P09’s father] being functional because he’s very concerned about appearances. And so, he definitely is the type of person to downplay anything.” The participant’s father exemplified how gender-based behaviors, such as the tendency to “downplay” issues and maintain an outward appearance of independence and high functioning (Affleck et al., 2018; Novak et al., 2019), can influence assessments of eligibility for formal support. This participant reported experiencing exasperation and additional stress as they attempted to access support for their father, only to have resources inadequately resourced when care providers did not consider his need to misrepresent support needs in determining care planning.

### Uncertainty

Uncertainty was a significant feature of the experiences of participants, yet it was not part of the original TIC framework. Participants consistently expressed feelings of uncertainty while accessing formal support for individuals living with dementia. This uncertainty stemmed from various sources such as the progression of the disease, the types of services needed, and changes in family dynamics. This new principle is distinct from others because it can be a natural response or experience for an individual accessing formal supports. However, if not managed properly, it can lead to negative experiences for these families. It is important to note that this sense of uncertainty persisted throughout the participant’s experience, from the diagnosis of the person living with dementia to the phase where the required care became inadequate at home.

Receiving a diagnosis did not alleviate uncertainty for participants. Instead, they often felt the need for additional support to cope with the changes accompanying the dementia diagnosis. P06 expressed, “Having a diagnosis did absolutely nothing for us.” Many participants stressed the lack of guidance in the early stages, emphasizing the necessity of dementia ambassadors or navigators to assist family caregivers in navigating the system post-diagnosis.

Furthermore, participants encountered uncertainties while grappling with the complexities of navigating the health care system post-dementia diagnosis. P10 mentioned, “Imagine dealing with this healthcare system at 90 years old or something, with a spouse or somebody who has this diagnosis. And you’re just completely overwhelmed. And you don’t even know where to turn.” Accessing formal support was indicated as particularly challenging among participants with differing abilities and capacities.

Moreover, uncertainty continued to influence participants’ experiences even when support from health care professionals was available. As expressed by P05, “we’re doing nothing positive to navigate a very difficult journey for them [individual living with dementia]. Even if you had a doctor and stuff.” Participants stressed the need for more comprehensive support beyond biomedical approaches to care, such as focusing solely on symptom management, especially as the disease progresses. Uncertainty arose from a lack of information and resources for next steps during the participant’s journey, leading to feelings of loss and an overwhelming sense of uncertainty when these supports were absent.

## Discussion

Our research explores the experiences of family caregivers accessing formal support for individuals living with dementia through a TIC lens. We identified numerous instances where the needs for TIC were either met or unmet, aligning with existing TIC principles. In addition, we identified the principle of uncertainty, which may be unique to the dementia journey given the disease’s dynamic progression over time.

Our study underscores the significance of establishing and maintaining trusting relationships between families and health care teams, creating safety throughout caregivers’ interactions with the formal care system. Trusting relationships can lead to better communication and improved care outcomes (Tuijt et al., 2021). We found that caregivers’ trust can be eroded by disease-focused rather than person-centered approaches, inconsistency, lack of transparency about services, and insufficient acknowledgment of caregivers’ own needs. Conversely, trust was enhanced through positive interpersonal dynamics and recognition by formal service providers. These findings align with Bergmann et al. (2022), who emphasize the critical need for professional support for individuals with dementia and their informal caregivers, necessitating a trusting relationship between these parties. Bergmann et al. (2022) identify a trustworthy relationship, characterized by proactive, early, and ongoing communication, as essential for effective collaboration between health care teams and families. Such relationships also enhance the utilization of support services for families (Bergmann et al., 2022). Formal service providers must strive to establish trust among the families they serve to support efficient and effective service provision, improve caregiver mental health outcomes, and prevent re-traumatization.

Our findings indicate that care delivery approaches that disregard caregivers’ preferences and backgrounds contribute to care-related trauma. This corresponds with existing research demonstrating that dehumanizing health care practices, which overlook families’ socioemotional and practical needs, can be traumatizing for both persons with dementia and their caregivers. Events such as the lockdown policies during the COVID-19 pandemic—which restricted access to essential family support for persons in long-term care—led to significant emotional and psychological stress. These events highlight the necessity of creating a collaborative health care system that empowers informal caregivers and involves them in decision-making (Chu et al., 2022; Cooke et al., 2023). Caregivers frequently do not receive support that considers their unique needs and circumstances, such as personal health issues or obligations (Feldman et al., 2021). Formal supports are often implemented in a one-size-fits-all approach that focuses primarily on the needs of the person living with dementia, overlooking the strengths and challenges of families. Opportunities for regular participation in the formulation of health policies and being informed about care practices, plans, and guidelines can empower caregivers, give voice to their needs, and ensure their preferences are considered in the planning and delivery of care services (Chu et al., 2022; Couzner et al., 2022).

Furthermore, our findings demonstrate that collaborating with individuals or organizations to achieve a shared objective exemplifies power-sharing in action. This promotes a sense of balance, reciprocity, and shared understanding, emphasizing the significance of each person’s roles and contributions. Our results resonate with existing research indicating that the formal supports system often fails to engage caregivers as partners and experts in care (Häikiö et al., 2020; Singh et al., 2014). In addition, we observed that connecting caregivers with others who have comparable life experiences can provide emotional, informational, and practical support not readily available through professional support groups. Our research indicates, consistent with other studies, that peer support for caregivers of individuals with dementia should be multifaceted (Carter et al., 2020). Effective peer support and interventions must encompass various aspects that families might need and be able to access. According to Carter et al. (2020), these components should include sharing information, developing skills, enhancing personal coping abilities, and providing support from both health care and non-health care professionals, as well as self-management strategies.

Importantly, we discovered that acknowledging sociocultural positioning, such as gender, is crucial for providing comprehensive formal support for caregivers and families of persons with dementia. This aligns with research indicating that gender differences significantly affect caregivers’ health (Martínez-Santos et al., 2021; Peacock et al., 2020). Specifically, women are more involved in hygiene and skin care for persons living with dementia and often neglect their own needs, leading to greater disturbances in health, such as rest and sleep, compared with men (Martínez-Santos et al., 2021). Caregivers’ experiences suggest that current policies and procedures for accessing formal care may lack specific considerations of gendered norms and expectations related to dementia care.

While caregiver experiences are shaped by sociocultural factors, their experiences also intersect with another critical aspect of dementia care: the unpredictability of the disease progression. This intersection highlights the need for a more nuanced understanding of the caregiving journey. Our study enhances existing conceptual work on TIC by demonstrating that the caregiving journey is not linear but dynamic and filled with uncertainty. Caregiving trajectories among persons living with dementia and their families include stages from disease diagnosis and ongoing symptom management to navigating family dynamics and future care needs when home support is no longer sufficient. Previous research indicates that a diagnosis can provide patients and their families with a sense of security and serve as a gateway to professional assistance (Sideman et al., 2023). However, our findings align with other studies that show caregivers receive insufficient support and resources from the formal health care system at the stage of disease diagnosis (Jameson et al., 2020; Mansfield et al., 2022). This lack of support hinders caregivers’ understanding of dementia and their ability to adapt care as the disease progresses and family needs evolve.

Uncertainty about dementia prognoses and available supports and services can lead to anxiety and burnout among caregivers (Laparidou et al., 2019; Parkman, 2022; Sideman et al., 2023). Our findings, along with those of previous studies, indicate a need to re-examine current assessment guidelines and referral procedures for formal support services to recognize the non-linear progression of families caring for persons with dementia. Effective monitoring practices that follow each family as their needs change and they navigate the formal support system can prevent trauma arising from unmet support needs.

These perspectives represented in this study reinforce the need for health care professionals to implement a trauma-informed approach when interacting with family caregivers of individuals living with dementia. Despite population aging and the increasing prevalence of dementia, there is a lack of research investigating TIC in gerontological care. Most studies on trauma experiences focus on veterans and war survivors (Isobel et al., 2021; Palfrey et al., 2019; Wilson et al., 2017), leaving gaps in understanding how older adults and caregivers accessing formal services experience or mitigate trauma. TIC plays a vital role in acknowledging caregivers within the system, addressing trauma from medical interactions, and transitioning toward a relational rather than biomedical model of care (Robertson et al., 2024). Implementing TIC in support services enhances communication, reduces disease-related stigma, and empowers caregivers through their interactions with care providers. Our study provides an important conceptual and practical contribution to the knowledge of TIC provision for families supporting individuals with dementia. Table 1 presents a set of adapted TIC principles, along with the clinical implications for practitioners seeking to apply them in health care settings.

There are limitations to this study. Most participants were women, and there was limited diversity in terms of income and ethnicity. The composition of the sample may have influenced how the principles of TIC were experienced. Although reminders and supports were provided (e.g., payment for time, coverage of costs for hiring care assistance to facilitate writing time), variations in the completion rates of diary entries were observed among participants. Consequently, some TIC-relevant experiences may not have been fully captured.

Considering the implications for family nursing, nurses should adopt a TIC approach when working with family caregivers and individuals living with dementia. An essential aspect of TIC is recognizing signs of trauma in families so they may be adequately supported. Signs such as anxiety, anger, and poor compliance with care plans may indicate lasting impacts from previous interactions with the health care system (Dowdell & Speck, 2022; Isobel & Edwards, 2017). Moreover, health care professionals should not assume “expert” roles, believing they fully understand and can identify the support needs of family caregivers and individuals living with dementia without input from these families. Intentional reflection and communication with families by nurses and health care professionals can help overcome personal biases and the misconception that all caregivers have similar experiences while navigating the journey of accessing formal care supports.

For meaningful TIC changes to occur, nurses and health care professionals must consider the power dynamics between themselves and family caregivers. Engaging in power-sharing activities with these families includes empowering caregivers with tools and resources to design their own care and working in partnership with them to support informed care decision-making. Although interactions with the health care system can be traumatizing for families, engaging in open and respectful communication with family caregivers to address their concerns and uncertainties about accessing formal support can foster trust, thereby supporting better care experiences and outcomes.

## Conclusion

Family caregivers are deeply involved in the care of individuals living with dementia. The impacts of caregiving on physical and psychological health, combined with traumatic experiences from interactions with the formal system, underscore the need to acknowledge caregivers’ contributions and integrate their expertise in designing care services. Tailored supports are needed that address the physical and mental health needs of caregivers throughout their caregiving journey. The exploration of family caregivers’ experiences through a trauma-informed lens remains limited in nursing research. This gap in knowledge affects our understanding of how nursing can effectively address the needs of older adults and their caregivers using a TIC approach. As the population ages and caregiver demographics diversify, it is crucial to meet the expanding needs of all caregivers. Developing a care system that reduces feelings of insecurity and uncertainty among families, enhances communication and collaboration across caregivers and providers, and shares power across these groups will reduce the likelihood and impacts of trauma among caregivers and persons with dementia.
